# Selections

**Published:** 1910-07

**Authors:** 


### SELECTIONS

---

### DIVISION OF FEES.

It has been said that it is the heaven-born privilege of an editor to talk about reform. Under this dispensation we call attention to the clandestine yet notorious bargaining carried on in the profession by those who have not the countenance to openly profess what they actually practice.

Splitting fees, the paying or receiving commissions for patients referred, is a reprehensible traffic that unquestionably victimizes unsuspecting patients, discredits trusted participants and degrades an honorable profession.

That this brokerage in patients is carried on is beyond denial —to what extent is for apparent reason difficult to determine, however, the exposure of the decoy letter correspondence in the

Chicago Daily Tribune, some years ago gives convincing indication of its prevalence. These letters, framed by a Chicago specialist for the purpose of collating the replies and reporting them to a local society, were sent from Odell, Ill., to 100 Chicago surgeons and physicians and read as follows:

"My Dear Doctor:

I have a case under my charge which requires attention along your line of work. I am not quite sure of my diagnosis, but will leave that whole matter to you. The people have heard of you favorably and are inclined to go to you for treatment. As they are wealthy they ought to pay a good fee for the service. Now, doctor, you understand that I am a young man just starting a practice, and in small towns we cannot make any distinction between the rich and poor in matters of charges. I have only received my regular visiting fee in this case. I understand, however, that it is customary for physicians to pay a commission of 25 per cent. upon all referred work, and I will deem it a favor if you can take care of me in this matter.

Kindly let me hear from you by return mail, as I am anxious to bring the case to you at once if satisfactory arrangements can be made.

Yours truly,"

---

Of the forty-four replies eighteen consented to the arrangement—a percentage that staggers belief; unfortunately (?) the entire correspondence fell into the hands of the lay press, which published the letters of those accepting—and lo! the names of the prominent were there.

But this infectious evil that thrives under cover is neither sporadic in Chicago nor endemic in this country. We learn that the eminent Pean was a pillar in this subterranean institution of commercial surgery in France; that Louis XIV. was on one occasion phlebotomized by an ambitious young surgeon, recommended by the King's Physician and when it was discovered that the latter had profited by the division of the fee paid the operator, the Council of State unanimously voted his death for "having made traffic of royal blood." It has recently come to light that representative members of the German profession have been bartering patients. In Belgium the Council of Physicians have had the matter before them for discussion and have given this buying and selling of patients the name of dichotomy.—But it does not matter by what technical name it is disguised, or how cunningly it is carried on or how skillfully concealed from the patient, it is the same everywhere—graft, pure and simple.

Surely the general practitioner should be paid for the services he has rendered, but why commission the specialist his collector in secret and in low breath? If any compact is made at all it should be made openly with the previous consent of the patient. The family physician has no right to receive compensation save for professional services rendered by him and it is unconscionable to secretly profit because his patients need more expert service than he can give.

Nothing is calculated to more speedily bring the honorable profession to greater disrepute than the layety's knowledge of this illicit practice. The exaction of money for one purpose and its surreptitious diversion to another is not a mere dishonesty but a breach of confidential relation so sacred as to make the suppression of the truth an act of disloyalty.

"No man can serve two masters,"—he cannot serve both his Divine Art and Mammon. The surgeon who will split fees will increase the size of his bill to keep himself harmless from loss, and the family physician from self interest, will be tempted to overlook the overcharge, and thus the *uberrima fides* existing from time immemorial between patient and doctor will be forever destroyed.

But how to eradicate this underhand "dickering" is the problem. Medical societies can do no more than stamp their disapproval upon it; they can no more control it than they can the abortion evil, for the dark secrecy of these shady transactions prevent detecting who's who. No medical legislation can enforce honesty,—for in the last analysis the lack of honest candor is the gist of the wrong.

The suggestion, that medical colleges should teach students what is ethically right, raises the question whether all professors are like Cæsar's wife—above suspicion and reminds one of Satan rebuking Sin.

Very recently the following resolution was passed by the Indiana University School of Medicine:

"*Resolved,* That any member of the faculty or teaching staff of the Indiana University School of Medicine, who shall be shown to be guilty, either directly or indirectly, of fee splitting, making an offer to split a fee, paying a commission for patients referred, or any violation of Article 6, Section 4, of the Principles of Medical Ethics of the A. M. A., shall be considered as having so impaired his usefulness as a member of the faculty or teaching staff of the School of Medicine by such unethical example to students, as to make his further connection with the faculty undesirable."

The adoption of this resolution by all medical colleges will be a stride in the right direction and will afford an unmistakable ethical precept to the student of medicine.—*Ed. American Practitioner and News.*

